# Phylogenetic Position and Subspecies Divergence of the Endangered New Zealand Dotterel (*Charadrius obscurus*)

**DOI:** 10.1371/journal.pone.0078068

**Published:** 2013-10-25

**Authors:** Julia M. I. Barth, Michael Matschiner, Bruce C. Robertson

**Affiliations:** 1 Department of Zoology, University of Otago, Dunedin, New Zealand; 2 Allan Wilson Centre for Molecular Ecology and Evolution, Department of Mathematics and Statistics, University of Canterbury, Christchurch, New Zealand; University of Sydney, Australia

## Abstract

The New Zealand Dotterel (*Charadrius obscurus*), an endangered shorebird of the family Charadriidae, is endemic to New Zealand where two subspecies are recognized. These subspecies are not only separated geographically, with *C. o. aquilonius* being distributed in the New Zealand North Island and *C. o. obscurus* mostly restricted to Stewart Island, but also differ substantially in morphology and behavior. Despite these divergent traits, previous work has failed to detect genetic differentiation between the subspecies, and the question of when and where the two populations separated is still open. Here, we use mitochondrial and nuclear markers to address molecular divergence between the subspecies, and apply maximum likelihood and Bayesian methods to place *C. obscurus* within the non-monophyletic genus *Charadrius*. Despite very little overall differentiation, distinct haplotypes for the subspecies were detected, thus supporting molecular separation of the northern and southern populations. Phylogenetic analysis recovers a monophyletic clade combining the New Zealand Dotterel with two other New Zealand endemic shorebirds, the Wrybill and the Double-Banded Plover, thus suggesting a single dispersal event as the origin of this group. Divergence dates within Charadriidae were estimated with BEAST 2, and our results indicate a Middle Miocene origin of New Zealand endemic Charadriidae, a Late Miocene emergence of the lineage leading to the New Zealand Dotterel, and a Middle to Late Pleistocene divergence of the two New Zealand Dotterel subspecies.

## Introduction

According to the IUCN Red List, the New Zealand Dotterel, or New Zealand Plover (*Charadrius obscurus*), is an endangered species and thus facing a very high risk of extinction in the wild [1]. These relatively large shorebirds of the family Charadriidae are endemic to New Zealand (NZ) and have not yet been molecularly placed in a phylogeny with their allies. Since morphological, biochemical and molecular phylogenetic investigations disagreed on the relationships of shorebirds (*Charadriiformes*), several studies using a diverse choice of genetic markers have attempted to solve the shorebird phylogeny [2]–[5]. However, these studies and a supertree approach focusing on the phylogeny of Charadriiformes left most nodes within Charadriidae unresolved [6]. Allozyme and cytochrome b variation within Charadriidae were examined with only a selection of taxa [7], [8], and suggested that *Charadrius* is paraphyletic by inclusion of the genus *Vanellus*. This demonstrates the need for a better resolved and more comprehensive phylogeny in order to resolve the position of taxa assigned to the genus *Charadrius*, including the New Zealand Dotterel.

The NZ endemic *Charadrius obscurus* is divided into two subspecies, breeding in two geographically widely separated locations between the upper part of the NZ North Island, mostly on the east coast north of 39°S, and Stewart Island, located south of the NZ South Island [9]. In 2004, only about 1700 individuals of the northern subspecies (*C. o. aquilonius*) were counted, which is nationally classified as Vulnerable by the New Zealand Department of Conservation [9], [10]. In contrast, the southern subspecies (*C. o. obscurus*) numbered just 250 individuals in 2005 and is nationally classified as Critical [9], [10]. Both populations were once widespread over the islands but declined dramatically during the last 150 years as a result of European settlement and introduced predators [9], [11]. Since the first recording of population sizes, the northern group always numbered more birds than the southern group, with growing numbers for both populations since intensive management started [9], [12]. The southern population experienced a serious bottleneck since the 1950s, which reduced the number of individuals by as much as 82%, and left only 62 surviving birds in 1992 [13]. Most of the birds were lost to introduced predators including feral cats and rats, which preyed mainly on the nocturnal-incubating males, leading to their increased shortage within the already minuscule population [14].

There is little written record or sub-fossil material to reconstruct the distribution of the New Zealand Dotterel before the 20th century [15]. Walter L. Buller wrote in 1888 that the bird is “dispersed along the whole of our shores” ([16], page 209) and specimens on the South Island were found as far north as Nelson, along the Southern Alps, close to Christchurch and near Invercargill [17]. There are no records of northern birds on the South Island; however, some birds showing the behavior of the southern subspecies were recorded in the central North Island [15], [18], [19]. Unlike other migrating members of the Charadriidae family, the New Zealand Dotterel seems to be sedentary within its territory and does not move long distances [20]. There is no historical evidence that the two populations ever interbred, and the question of when and where the two subspecies diverged is still open [13]. However, it has been speculated that the separation occurred at or north of Cook Strait and that “recent free interbreeding between the two groups was less likely than longer isolation” ([15], page 230).

There are substantial morphological and behavioral differences between the northern and southern subspecies. Southern birds are larger in nearly all measurements, have darker plumage and breed inland above 300 m a.s.l., whereas North Island birds breed on sandy beaches or dunes, always very near the coast [15]. These remarkable differences were the reason for the description of the two populations as separate subspecies [15] and raise the question of whether the observed isolation is also reflected at a molecular level. Indeed, this point was addressed previously by Herbert and coworkers [21], who performed an allozyme analysis to evaluate the genetic variation of the two populations. However, no genetic difference could be detected between the northern and southern population, which the authors suggested might be due to the low resolution of this technique or a general low diversity within birds [21]–[23]. However, the geographical separation, as well as the distinct morphological and behavioral differences, warrants a revisit of the question of molecular separation between New Zealand Dotterels. This question is particularly important because both are endangered and threatened by different hazards and in different environments, thus necessitating an individually adapted conservation management scheme [9]. A clear genetic distinction between the subspecies would substantially facilitate this management.

In this study, we approach three questions: (1) what is the phylogenetic position of *Charadrius obscurus* within the family Charadriidae; (2) when did the northern and southern populations separate; and (3) what is the level of genetic differentiation between the two subspecies *C. o. obscurus* and *C. o. aquilonius*? To answer these questions, we conducted phylogenetic analysis of mitochondrial and nuclear DNA markers.

## Materials and Methods

### Ethics Statement

Samples were collected by and under the permit of the New Zealand Department of Conservation.

### Sample Collection

Blood samples of a total of 14 *C. obscurus* individuals were sampled. Seven *C. o. aquilonius* (northern population) individuals were collected along the Bay of Plenty, North Island, New Zealand at the following locations: Herepuru, Pikowai, Pukehina, Hauone, Maketu Spit and Matakana Island. Blood samples of seven *C. o. obscurus* (southern population) individuals were collected at Awarua Bay, South Island, New Zealand, as well as in Mason Bay and on Table Hill, Stewart Island, New Zealand.

### DNA Isolation, Amplification and Sequencing

Genomic DNA was extracted from 10–20 µl blood using 400 µl 5% chelex-100 resin solution plus 5 µl proteinase K during an incubation at 55°C for 4 hours. DNA was precipitated using 0.05 M lithium chloride in 100% ethanol. Amplification of the mitochondrial cytochrome b gene (*cytb*, 1143 bp, primer pair L14764 and H16064, [24]) and the 7th intron of the nuclear beta-fibrinogen gene (*bFI7*, 936 bp, primer pair FIB-B17U and FIB-B17L, [25]) was performed for all 14 individuals on an Eppendorf Mastercycler pro as described elsewhere [26]. The mitochondrial control region (*CR*; 1008 bp) was targeted for six *C. o. aquilonius*, and three *C. o. obscurus* individuals using the primer ND6F (5′- CCC TAA AAA AAG CAC AAA ATA AGT CAT), which binds at the 3′ end of *ND6* and tRNA-PheR (5′-CTT GGC ATC TTC ATT GCC ATG C), which binds within the *tRNA-Phe* sequence. Primers used to amplify a part of the mitochondrial *12s rRNA* gene (267 bp) from one *C. o. aquilonius* individual were: cytb1051F (5′- ATC GGC CAA CTA GCC TCC CTC AC) and 12s554R (5′- GGC ACC GCC AAG TCC TTA GAG). Amplification volume was 25 µl, containing app. 50 ng of template DNA, 0.2 mM each of dNTP, 1.6 mM MgCl2, 0.4 µM of each primer and 1 unit BIOTAQ polymerase (Bioline, London, UK). For amplification of the *CR*, cycling parameters were an initial 5 min denaturation at 95°C, followed by a touchdown of 17 cycles at 95°C/25 sec, 64°C to 55°C/30 sec and 72°C/2 min and 20 cycles at 95°C/25 sec, 55°C/30 sec and 72°C/2 min, ending with a final 5 min 72°C incubation. The protocol for amplifying *12s rRNA* was an initial 95°C/5 min, 45 cycles at 95°C/25 sec, 64°C/30 sec, 72°C/2 min and a final incubation for 5 min at 72°C.

All amplicons were examined by agarose gel electrophoresis, reactions were purified with multi-well filter plates AcroPrep Omega 30 K (PALL Corporation, Port Washington, NY, USA) and PCR fragments were sequenced using L14764 and H16064 primer for *cytb*, FIB-B17L for *bFI7*, ND6F and tRNAPheR for the *CR* and 12s554R for the *12s rRNA* gene on an ABI 3730xl DNA analyser (Applied Biosystems, Carlsbad, CA, USA) operated by the Genetic Analysis Services, University of Otago, New Zealand. Sequences were aligned and edited using Geneious v6.0.4. (Biomatters, New Zealand) with default settings. All sequences are deposited under the following GenBank accession numbers: KF357966-KF357995.

### Phylogenetic Analyses and Molecular Dating

To infer the phylogenetic placement of *Charadrius obscurus* within the family Charadriidae, we used three approaches: a maximum likelihood (ML) and two different Bayesian inference (BI) methods, all with partitioned data sets. Available mitochondrial and nuclear DNA sequences for members of the Charadriidae family and one outgroup (Haematopodidae) were retrieved from GenBank and The Barcode of Life Database [27], [28]. All DNA markers used are listed with accession numbers in Table S1. Individual markers were aligned using the default settings in MAFFT v7.029b [29] and visually checked and corrected using Mesquite v2.75 [30]. We tested for phylogenetic congruence between markers with the software Concaterpillar v1.7.2 [31], whereby all mitochondrial sequences were considered as a single marker, and the two nuclear markers *bFI7* and *RAG1* were used separately. As only three taxa had sequence data available for both *bFI7* and *RAG1* (see Table S1), we could not run a single Concaterpillar analysis with mitochondrial sequences, *bFI7*, and *RAG1*. Instead, we ran two individual analyses to test for incongruence between mitochondrial sequences and each of the two nuclear markers. Congruent datasets were concatenated, again using Mesquite [30]. In order to account for different substitution models, we applied data partitioning and grouped by coding position (cp) and molecule type (mitochondrial/nuclear). Non-coding markers were considered as individual partitions. The partitions were as follows: cp1, cp2, cp3 of all coding mitochondrial genes (*cytb*, *ND2*, *ND3*, *ATPase6*, *ATPase8*, *CO1*), cp1, cp2, cp3 for *RAG1*, non-coding mitochondrial sequences (*12s* and *16s*, separated) and non-coding nuclear DNA (*bFI7*). The best-fit models of nucleotide evolution (Table S2) were selected according to the Bayesian Information Criterion (BIC) in jModelTest v2.1.1 [32]. An ML search with 10 individual runs to find the tree with the best likelihood score and a run with 1,000 bootstrap (BS) replicates were performed using GARLI v2.0 [33] on the CIPRES Science Gateway [34], beginning with a stepwise-addition starting tree (“attachmentspertaxon”  = 50) and applying the termination conditions “genthreshfortopoterm”  = 20,000 and “scorethreshforterm”  = 0.001. We repeated the ML search with random starting trees, which resulted in the same topology. To perform the BI analysis, we used MrBayes v3.2 [35] with 1,000,000 generations per run and four parallel Monte Carlo Markov chains (MCMC). AWTY [36] was used to assess chain convergence. After discarding the first 25% of MCMC generations as burn-in, tree topologies were summarized and the consensus tree was visualized using FigTree v1.4 (http://tree.bio.ed.ac.uk/software/figtree).

Given the very poor fossil record of the family Charadriidae, time calibration was based on age constraints resulting from a recent large-scale molecular dating of extant birds [37|. As the basal topology of Charadriidae disagrees between individual molecular phylogenies (e.g. [4], [37]), we chose to adopt an age constraint for the most ancient well-supported internal node rather than the root. Thus we constrained the age of the most recent common ancestor of all Charadriidae except *Pluvialis* according to the results of Jetz et al. [37]. In order to reflect these results, a normally-distributed prior was assigned for this age constraint with a mean at 38.85 Ma and a soft minimum and maximum age of divergence between 46.3 – 31.4 Ma.

Time-calibrated phylogenies were estimated with BEAST v2.0.2 [38], [39]. All BEAST runs were performed using mitochondrial and nuclear sequence alignments as separate partitions with unlinked substitution models. Substitution models were evaluated by an automatic model selection and averaging approach newly implemented in BEAST 2 [40]. We employed a relaxed molecular clock model with branch rates drawn independently from a lognormal distribution [39], one time constraint (see above), and the reconstructed birth-death process [41] as a tree prior. We performed three independent analyses of 50,000,000 generations each, discarding the first 5 million generations of every replicate as burnin. Replicate results were combined in LogCombiner v2.0.2 (http://beast.bio.ed.ac.uk/LogCombiner) and convergence of run replicates was confirmed by effective sample sizes (ESS) >200 for all parameters and by visual inspection of traces within and between replicates using Tracer v1.5 [42]. The resulting posterior sample of trees was summarized in a Maximum Clade Credibility (MCC) tree using TreeAnnotator v.2.0.2 (http://beast.bio.ed.ac.uk/TreeAnnotator). Garli BS and MrBayes Posterior Probability (PP) values were mapped onto this MCC tree using SumTrees v3.12.0 [43]. Graphics were processed using Adobe Illustrator CS5 (http://www.adobe.com).

### Genetic Structure Analysis

The mitochondrial gene *cytb* (1143 bp) and the nuclear intron *bFI7* (936 bp) were analysed for all *C. obscurus* individuals (seven specimens of *C. o. aquilonius* and seven specimens of *C. o. obscurus*). In addition, we analysed the *CR* (1008 bp) of six northern and three southern samples and verified the sequences obtained by comparison with other *Charadrius CR* sequences and an annotated *CR* sequence of *Phoebastria albatrus* (GenBank acc. no. AB254201) [44]. The genetic structure of *cytb* and *bFI7* genetic sequences among all individuals was analysed using STRUCTURE v2.3.4 [45], testing for the presence of 1–4 genetic clusters (*K* = 1–4), and using a burn-in of 10,000 of a total 100,000 MCMC generations. Analysis of molecular variance (AMOVA; [46]) and calculation of pairwise fixation indices (F_st_) values was performed using ARLEQUIN v3.5 [47]. In order to illustrate the genetic structure of *Charadrius obscurus*, we used PopART v1.1 beta (http://www.leigh.net.nz/software.shtml) to create a median joining haplotype-network of the *cytb* sequences.

## Results

### Charadriid Phylogeny and Molecular Placement of the New Zealand Dotterel

In order to infer the phylogenetic placement of the New Zealand Dotterel (*Charadrius obscurus*) within the family of Charadriidae using molecular data, we sequenced mitochondrial (*cytb*, *12s*) and nuclear sequences (*bFI7*). Additionally, we retrieved one mitochondrial sequence (*CO1*) from a publicly accessible database (Table S1). For other members of this family (altogether 40 taxa) and three outgroup taxa, we retrieved sequences for a total of ten markers, including eight mitochondrial and two nuclear markers, from publicly accessible databases (see Table S1 for accession numbers). The *CR* sequences were excluded from the phylogenetic analysis since the interspecific *CR* data could not be aligned with confidence. After removal of gaps, the total length of the aligned data set covered 9731 bp within 43 taxa, comprising 66% missing sequences or 70% missing characters. According to hierarchical likelihood-ratio tests with the software Concaterpillar [31] no phylogenetic incongruence was detected between mitochondrial sequences and either *bFI7* (*p*-value 0.44) or *RAG1* (*p*-value 0.33).

**Figure 1:**
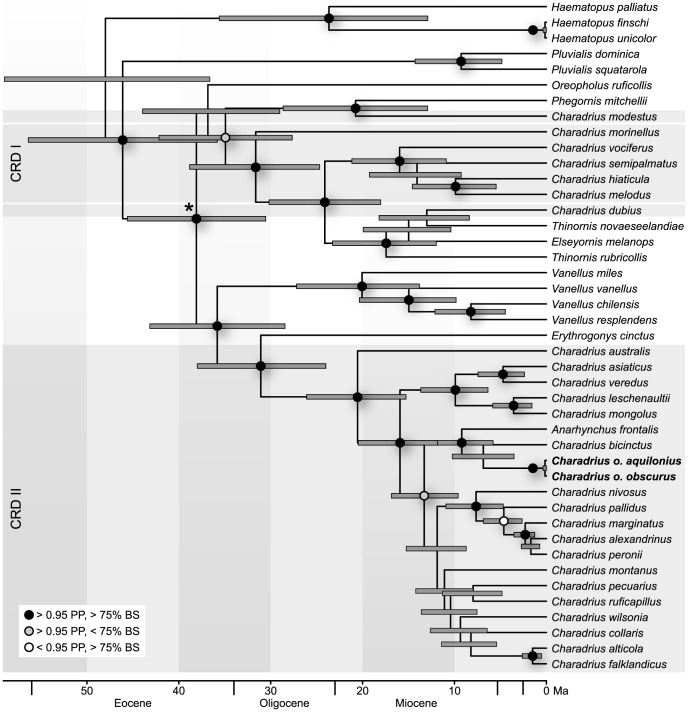
Placement of *C. obscurus* within a time-calibrated phylogeny of Charadriidae. Shown is the BEAST topology. Black dots indicate nodes with Bayesian Posterior Probability (PP) >0.95 and Bootstrap Support (BS) >75, grey dots indicate PP >0.95 and BS <75 and white dots PP <0.95 and BS >75. PP and BS values that are both lower than 0.95 and 75, respectively, are not indicated (for all support values, please see Fig. S1). Horizontal grey boxes (CRD I and II) highlight the non-monophyletic *Charadrius* groups. The asterisk marks the time-constrained split; node bars show 95% highest probability density (HPD).

The phylogenetic relationships recovered using maximum likelihood (ML) and Bayesian inference (BI) methods (Fig. 1 and Fig. S1) were largely congruent other than for *Oreopholus ruficollis*, which appears sister to all other taxa (except *Pluvialis* and *Haematopus*) in the MrBayes BI topology (PP 0.49), but is placed within this clade in the ML and BEAST BI analyses (BS 60, PP 0.93). Furthermore, inferred relationships within the genus *Charadrius* are inconsistent: ML and BEAST BI analyses resolve a group combining the closely related species *C. asiaticus*, *C. veredus*, *C. leschenaultii* and *C. mongolus* sister to a clade combining 14 taxa of *Charadrius* plus *Anarhynchus frontalis* (within the CRD II group, see below) (BS 64, PP 0.99) whereas MrBayes places *A. frontalis*, *C. bicinctus* and the *C. obscurus* subspecies (PP 0.55) at this position (Fig. S1). BS and PP values for the youngest clades of the genus *Charadrius* are not highly supportive, resulting in slightly different topologies (Fig. S1).

The MCC tree of BEAST received higher node support than the MrBayes topology, therefore we discuss it in more detail (Fig. 1). In agreement with earlier studies [5], the genus *Pluvialis* was recovered as the most basal group within the family Charadriidae. Members of the genus *Charadrius* appear to be non-monophyletic and cluster in two groups (named CRD I and CRD II in Fig. 1), although a well-supported monophyletic clade is formed by the combined genera *Charadrius*, *Phegornis*, *Vanellus*, *Anarhynchus*, *Thinornis*, and *Elseyornis*.

The CRD I group, which includes the species *Charadrius modestus*, *C. morinellus*, *C. vociferus*, *C. semipalmatus*, *C. melodus*, *C. dubius* and the type-species *C. hiaticula*, forms a strongly supported monophyletic clade together with *Thinornis* and the monotypic genera *Phegornis* and *Elseyornis*. Within this group, the two *Charadrius* species *C. modestus* and *C. dubius* are placed in clades with *Phegornis* and *Thinornis*/*Elseyornis*, respectively. The proposed position of *C. dubius* as the sister species of *T. novaeseelandiae* resulted from only 530 bp of the gene *CO1*, the only marker for which sequence information was available for both taxa. Thus, the clade combining the two species is weakly supported (BS 46, BEAST PP 0.55, MrBayes PP 0.49), whereas the position of *C. modestus* as sister to *P. mitchellii* receives strong support in all analyses (BS 100, BEAST PP 1.0, MrBayes PP 1.0).

The CRD II group includes all other *Charadrius* members of this study as well as the monotypic genus *Anarhynchus* and forms a strongly supported monophyletic clade with the Lapwings (genus *Vanellus*) and the monotypic genus *Erythrogonys*. The Red-kneed Dotterel (*Erythrogonys cinctus*) has previously been grouped with *Vanellus* in the subfamily *Vanellinae* [8], but here appears as the sister-clade to the CRD II group with strong support (BS 92, BEAST PP 1.0, MrBayes PP 1.0). Monophyly of the two subspecies of the New Zealand Dotterel (*C. o. obscurus* and *C. o. aquilonius*) was strongly supported (BS 100, BEAST PP 1.0, MrBayes PP 1.0). Both were recovered within the second group of *Charadrius* (CRD II), as sister clade to the Double-banded Plover (Banded Dotterel) *C. bicinctus* in the BI topologies (BEAST PP 0.63; MrBayes PP 0.58) and sister to the Wrybill *Anarhynchus frontalis* in the ML topology (BS 67) (Fig. 1 and Fig. S1). The three taxa together form a clade endemic to NZ [19], which is well-supported in the BEAST and ML tree (PP 0.97, BS 77) and recovered with lower support in the MrBayes topology (PP 0.60). In the BEAST and ML topologies, the NZ clade is sister to a poorly resolved clade of CRD II birds including a geographically variegated group of plovers and dotterels.

### Charadriid Divergence Date Estimates

The fossil record of Charadriidae is limited to fragmented remains, the taxonomic assignments of which have not been verified [48], [49]. In order to estimate divergence times for the New Zealand Dotterel, we therefore time-calibrated our phylogenetic data set with a constraint derived from a recently published large-scale phylogeny of extant birds, which was dated on the basis of ten well-known fossils (Fig. 1, asterisk: 46–31 Ma) [37]. Our results support an Eocene origin of Charadriidae (mean 48 Ma, 95% highest posterior density (HPD) 59–36.6 Ma), which approximates the average of the results of Paton, Thomas, Baker and coworkers [4], [6], [50]. The clade combining the three plovers endemic to NZ (*A. frontalis*, *C. bicinctus* and *C. obscurus*) apparently originated in the Middle Miocene (mean 13.3 Ma, 95% HPD 16.9 – 9.6 Ma). The separation of the lineage leading to the Wrybill (*Anarhynchus frontalis*) represents the first divergence event within this clade (mean 9.2 Ma, 95% HPD 12.9 – 5.8 Ma), before the split between the Double-banded Plover (*C. bicinctus*) and the New Zealand Dotterel about 6.9 Ma ago (95% HPD 10.2 – 3.5 Ma). The divergence of the two New Zealand Dotterel subspecies happened very recently (mean 150 ka, 95% HPD 370 – 3 ka). Thus, these three species most likely derive from one single dispersal event to NZ. As a fourth species endemic to NZ, the Shore Dotterel (*Thinornis novaeseelandiae*) was included in our phylogeny. The origin of the lineage leading to *T. novaeseelandiae* was estimated at about the same time as the other plovers endemic to NZ (mean 13.0 Ma, 95% HPD 18.2 – 8.4 Ma); however, this species was resolved within the paraphyletic CRD I group in our phylogeny and thus must have independently dispersed to NZ (Fig. 1).

### Subspecies Differentiation of *C. o. obscurus* and *C. o. aquilonius*

**Figure 2:**
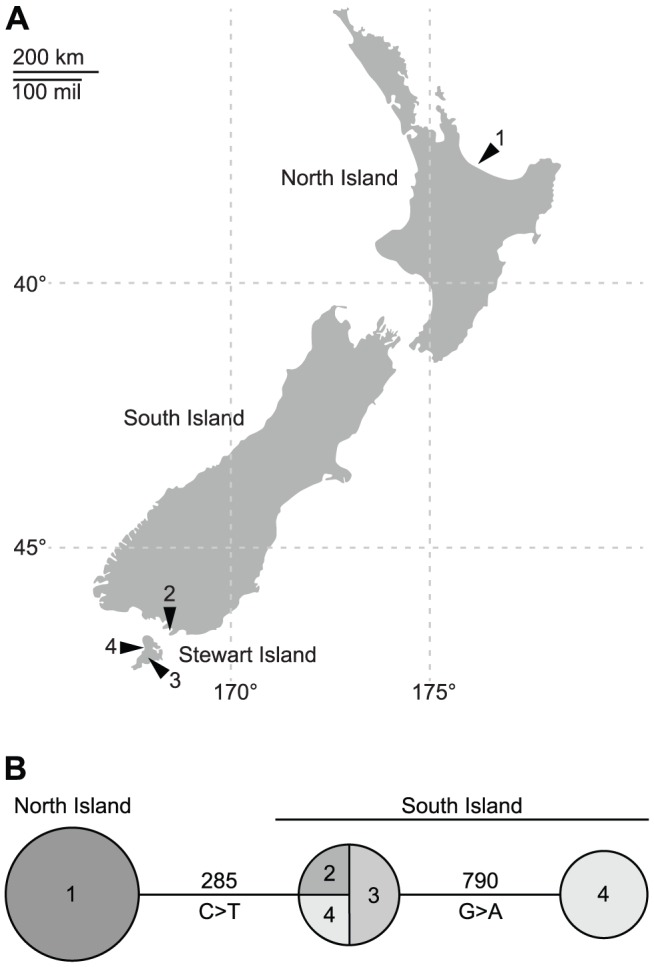
New Zealand Dotterel sample locations and haplotypes. A) Distribution of sample sites. Northern population: 1, Bay of Plenty (7 samples). Southern population: 2, Awarua Bay (1 sample); 3, Table Hill (2 samples); 4, Mason Bay (4 samples). B) Cytochrome b haplotype genealogy. Circle radius indicates quantity of individuals; numbers in circles refer to sample locations shown above.

The two endangered *C. obscurus* subspecies (*obscurus* and *aquilonius*) are geographically widely separated, not only by a distance of more than 1000 km, but also by the Cook Strait which poses a barrier to the dispersal of many NZ species [15] (Fig. 2A). We questioned whether this geographic isolation has led not only to behavioral and morphological differences [15], but also to a genetic diversification. We therefore sampled *C. o. aquilonius* from one location on the North Island and *C. o. obscurus* from three different locations on the South and Stewart Island (Fig. 2A), and sequenced two mitochondrial (*cytb*, *CR*) and one nuclear loci (*bFI7*). Verification of our *CR* sequences by alignment with a well-annotated *CR* sequence of *Phoebastria albatrus* revealed 70% identity (gaps 7%) and entirely identical characteristic features as the bird box and the E box [44]. Based on this alignment, our sequence spanned the entire *CR*, including conserved and variable parts but lacking the repeated 3′ part of the *P. albatrus* sequence. The *CR* as well as the *bFI7* sequences also aligned well with other *Charadrius CR* and *bFI7* sequences and no ambiguous areas were detected in the chromatograms. Translation of the *cytb* sequences revealed no stop-codons within the sequences.

Analysis of sequence variation showed surprisingly little diversity between the examined individuals in all markers. We found no inter- nor intra-populational variation at the 1008 bp *CR* locus. We also found consistently low levels of polymorphism within the *bFI7* and the *cytb* genes. Within 936 bp of *bFI7*, we detected four segregating sites (2 transitions, 2 transversions), resulting in two alleles, which are shared between both subspecies. Within the 1143 bp of the *cytb* gene, 2 transitions were detected, resulting in three haplotypes, which differ between northern and southern populations. Thus, the percentage of polymorphic sites was 0% for the *CR*, 0.4% for *bFI7* and 0.2% for *cytb*. Pairwise F_st_ values were calculated to measure the differentiation between both populations (0 indicating no divergence, 1 meaning complete separation) [51]. Whereas the F_st_ for the shared *bFI7* alleles yields a non-significant value of 0.0 (*p*-value 0.86), the F_st_ for *cytb* results in an F_st_ of 0.8 (*p*-value 0.001), indicating significant divergence between the populations. This result is illustrated in a haplotype genealogy, where all samples from the North Island are combined into one cluster, which is separated by a single substitution from two southern clusters (Fig. 2B). Within the southern individuals of *C. obscurus*, we found two haplotypes separated by one substitution that isolates three samples from Mason Bay from those collected on Table Hill, Awarua Bay and another fourth Mason Bay specimen (Fig. 2A and B), pointing to a possible substructure within the southern population. However, analysis with the program STRUCTURE revealed no substructure of the populations together or the southern population only.

## Discussion

### Charadriid Phylogeny and Molecular Placement of the New Zealand Dotterel

A molecular phylogeny combining the available genomic sequences to resolve the relationships within the family Charadriidae was previously not available. Here we used a partitioned dataset including mitochondrial and nuclear sequences of 26 out of 31 taxa currently assigned to the genus *Charadrius* as well as representative taxa of all other genera within Charadriidae in order to place *C. obscurus* within the shorebird phylogeny. To accommodate the heterogeneity of the diverse genetic data, we partitioned the alignment into groups of sites that are assumed to have evolved under similar processes, a method shown to improve phylogenetic estimations [52], [53]. A nearly species-level coverage of the genus *Charadrius* was essential to identify the monophyletic components of this apparently polyphyletic genus, and to correctly place *C. obscurus* within one of these subgroups. Therefore, we also included taxa with little available sequence data, which led to a high percentage of missing characters. Although some studies found that incomplete data could bias maximum likelihood (ML) and Bayesian inference (BI) analysis [54], other authors have argued that missing data has no corruptive effect on the accuracy of phylogenies in both ML and BI analysis, and that the addition of characters typically improves phylogenetic accuracy even if much of the information for these characters is missing [55], [56].

Most members of the genus *Charadrius* are recovered within two groups (CRD I, CRD II), however, *C. modestus* and *C. dubius* cluster with *Phegornis* and *Thinornis*/*Elseyornis*, respectively. In a previous study surveying *Charadrius* relationships using *cytb* sequences, *C. modestus* appeared in a clade with members of our CRD I group (*C. semipalmatus*, *C. vociferus*) and *Thinornis*/*Elseyornis* [7]. Another study, using morphological and behavioral traits, found *C. modestus* in close association with the CRD II group [57], whereas our own molecular phylogenies place *C. modestus* and *P. mitchellii* in close relation with members of the CRD I group, thus corroborating the results of Joseph et al. [7]. However, more data will be necessary to verify this position. Non-monophyly of the genus *Charadrius* has been supported previously [7], [8], and the study of Joseph and coworkers [7] agrees with our phylogeny in finding the genus *Vanellus* more closely related to CRD II than to CRD I. Furthermore, our phylogeny agrees to a great extent with those of Joseph et al. and Phillips [7], [57] on taxon composition within the two main sub-groups of *Charadrius* (CRD I and CRD II), the only exception being the position of *C. modestus*. Phillips' sub-groups were characterized by morphological and behavioural differences: members of CRD I are known to have more eggs, chicks are boldly patterned, eyelids are colored, the mount time is brief, scrape is exchanged under tail of scraper instead of the side and they do not bow, tilt or “moo” as members of CRD II do [57]. Apparently in contrast to these and our results, a recent study by Livezey [58] based on phenotypic characters recovered the genus *Charadrius* as monophyletic. However, Livezey considered members of our CRD I group (e.g. *C. modestus* and *C. morinellus*) to be excluded from this genus, and to belong to the genera *Zonibyx* and *Eudromias* instead. Furthermore, most nodes within Charadriidae are poorly supported in this study, and a majority rule consensus tree of Livezey's phylogeny would not disagree with the intrageneric relationships recovered in our study.

According to our analyses, the Red-kneed Dotterel (*Erythrogonys cinctus*) is strongly supported as the sister taxon to the CRD II group, and both together appear as the sister of the genus *Vanellus*, which is in agreement with a recent publication on the phylogeny of Charadriiformes [4]. This relationship is surprising, since *E. cinctus* is considered part of the subfamily Vanellinae, to which it was assigned on the basis of protein allozyme similarities and shared morphological characters such as the retention of the hind toe, a feature that is not found in species of the genus *Charadrius* with the exception of *C. modestus* [8], [59]. Nevertheless, other relationships of *E. cinctus* with various subgroups of *Charadrius* have also been supported by morphological characteristics [57], [60]. Regardless of the exact relationship of *E. cinctus* with *Vanellus* and the CRD II group, our time-calibrated phylogeny suggests that the genus *Erythrogonys* separated from both of these groups as early as the Late Eocene or Oligocene.

Within the CRD II group, we identified the New Zealand Dotterel (*Charadrius obscurus*) as the sister taxon of the Double-banded Plover (*C. bicinctus*) and the clade combining these two species as the sister of the monotypic genus *Anarhynchus frontalis*. This matches their biogeographic distribution as all three species are endemic to NZ [19]. In addition, both the New Zealand Dotterel and *A. frontalis*, the Wrybill, do not migrate as far as most other species of *Charadrius*, with *C. obscurus* flocking in winter very close to its breeding sites and *A. frontalis* only migrating within NZ to wintering flocks in Northland [19]. However, *C. bicinctus* is not as sedentary as the New Zealand Dotterel or the Wrybill and migrates in winter to Tasmania, Australia and even some of the south-west Pacific Islands [19]. One of the behavioral traits that *C. obscurus*, *C. bicinctus* and *A. frontalis* have in common is choking, a habit that has not been described for any other member of the family [57]. These three species also share a similar courtship behavior, which distinguishes them from other Charadriidae [57]. The Wrybill, which is nested within the CRD II group in our topology, has previously been included in the genus *Charadrius* [17], [61], [62], but is currently placed in its own monotypic genus due to its unique asymmetric bill [63]. However, this taxonomic position has been questioned, as beside its unusual bill, it resembles members of the genus *Charadrius* in all respects [60], [64]. In conclusion, based on the molecular phylogenies in this study and supported by ML and BI analyses, we propose *C. obscurus* to be the sister of *C. bicinctus* and to form a monophyletic clade with *A. frontalis*.

### Charadriid Divergence Date Estimates

The fossil record of Charadriidae seems to be problematic [48], [49], but our estimated age of origin of this family in the Eocene is congruent with the age of the oldest putative charadriid fossil described to date [65]. The earliest record of the presence of small waders in the fossil fauna of NZ derives from an Early to Middle Miocene deposit in Otago, which would agree with our age estimate for the clade combining the three NZ endemics *C. obscurus*, *C. bicinctus*, and *A. frontalis*. However, insufficient fossil material exists to determine whether these bones indeed belong to genus *Charadrius*, or to the scolopacid genus *Calidris* [66].

Our phylogenetic tree was time-calibrated with divergence date estimates resulting from a higher-level phylogeny [37] in order to obtain a timeline of charadriid diversification that is independent of the family's problematic fossil record [48]. This higher-level phylogeny had been time-calibrated with ten avian fossils including *Morsoravis sedilis* from the Early Eocene [37]. Following Dyke and van Tuinen [67], the authors assumed *Morsoravis sedilis* to be a member of crown Charadriiformes, and correspondingly constrained the age of this group. However, more recent evidence suggests that *Morsoravis* may be part of the stem rather than the crown group of Charadriiformes, or that it may not be a member of the order at all [68]. In this case, the ages obtained by Jetz et al. [37] for charadriiform divergences may be overestimated which would bias the timeline of our study towards older ages. As a consequence, true divergence dates of Charadriidae might be younger than those inferred in our time-calibration and could match those estimated in previous studies on the basis of the younger, and less disputed Late Eocene *Nupharanassa tolutaria* [6], [50] (see also [48]).

Time-calibration of our phylogeny supports a Middle Miocene arrival of dotterels in NZ. Given that *C. australis*, an Australian endemic, is the first lineage to diverge within the CRD II group, while the sister lineage of this group, *E. cinctus*, is native to Australia, it seems likely that the early diversification of the CRD II group took place in Australia. If so, the NZ clade may have originated from a dispersal event from Australia across the Tasman Sea. The Middle Miocene has been marked by global cooling events, associated drops in sea level and changes in oceanic currents [69]. This period also coincides with the emergence of seamounts of the Lord Howe chain between Australia and NZ, including Capel Bank and Gifford Guyot, which today lie 55 m and 290 m below sea level, but were likely to have been exposed in the Miocene [70]. Besides the seamounts of the Lord Howe Rise, the Miocene reefal limestones of Norfolk Island suggest that parts of the Norfolk Ridge to the north-west of NZ could have been exposed as early as 20 million years ago during periods of heavy glaciation [71], [72]. Thus, such islands could have served as stepping stones for dispersal, reducing the minimum oversea distance from approximately 1800 to around 800 km. Furthermore, volcanic island chains were likely to have connected the north of NZ and New Caledonia during the Miocene with spacings as small as 50–100 km [73]. It is known that a long list of birds, other animals and plants have dispersed from Australia to NZ [74] and thus it may be speculated that the change in climate and emergence of islands between Australia and NZ facilitated charadriid dispersal.

Divergence of the *C. obscurus* subspecies was dated between 3,000 years ago and the Upper Pleistocene (365,000 years ago). The Pleistocene period was affected by glacial episodes: permanent snow and glacier extended towards the coast, reducing forested regions and limiting species habitats to isolated areas [75]. New Zealand Dotterel colonies could have been forced to these isolated areas on the South and North Island during the Pleistocene, limiting or preventing genetic exchange for a long time and only slowly extending breeding habitats to the former range on both islands that is known from historical records [11].

### Subspecies Differentiation of *C. c. obscurus* and *C. c. aquilonius*

The subspecies status of the northern and southern New Zealand Dotterel populations is supported by differing behavior, morphological traits and geographic distribution [15]. However, a previous allozyme-based study was unable to demonstrate genetic differences between the two populations [21]. Here we have revisited the population structure of the New Zealand Dotterel using highly variable genetic sequence markers. In many avian species, the mitochondrial control region (*CR*) is one of the fastest-evolving molecular markers [76] and has been shown to evolve faster than cytochrome b (*cytb*) in at least one charadriiform genus [77]. Surprisingly, comparison of the *CR* between *C. obscurus* individuals revealed no genetic variation within each population, which could reflect a recent bottleneck event [78]. However, very low intraspecific diversity has also been described for other Charadriiformes of the genus *Larus*, suggesting slow rates of evolution for the *CR* [22]. Another possible explanation for the lack of variation is accidental amplification of slower evolving nuclear homologues (numts) instead of the mitochondrial *CR* [79]. Although we used specific primers, our sequences possess characteristic *CR* features, are devoid of heterozygous positions that would be indicative of a nuclear origin, and appear most similar to charadriiform *CR* sequences in BLAST searches, the amplification of nuclear copies can not be ruled out with certainty. We therefore regard this result with caution.

In contrast, unique haplotypes were observed among the northern and southern populations in the mitochondrial *cytb* gene, which is commonly thought to evolve more slowly than the *CR*. Sequence divergence of *cytb*, although limited, clearly separates the two populations and suggests genetic isolation. A divergence rate of 2% per million years is often used as a rough standard molecular clock for the *cytb* gene, though it has been shown that birds generally evolve slower than mammals, with Charadriiformes having a particularly slow substitution rate, even among birds [80]. According to rate estimates of Nabholz and coworkers [80], and applying the rate conversion proposed by the same authors, the per lineage substitution rate of charadriiform *cytb* sequences is ∼0.65% per million years. Taking this into account, the northern and southern subspecies could have diverged ∼135,000 years ago, a value that is close to the mean age estimate resulting from our BEAST analysis of 150,000 years.

Within the southern population we observed two *cytb* haplotypes isolated by a single transition. Whereas one of the two haplotypes was present at the three southern sampling sites, the other was private to Mason Bay. This might indicate a possible substructure with birds from Mason Bay nesting not at Table Hill but in other, unsampled, breeding areas on the island. According to Dowding and Murphy [13] *C. o. obscurus* adults are faithful to their flocking sites, however no correlation has been observed between flocking and breeding places. Thus, regardless of the actual breeding sites of birds sampled at Mason Bay, we expect that their haplotype is present in the other southern locations, but is missing in our data set due to our small sample size.

Since mitochondrial genes are linked, strictly maternally inherited, and do not generally undergo recombination, we also included nuclear markers in our data set [81]. The nuclear beta-fibrinogen intron 7 (*bFI7*) has previously been demonstrated to be a suitable marker for phylogenetic analyses of recently evolved species [82]. However, in the New Zealand Dotterel, the resolution of *bFI7* is too low to uncover differences between the northern and southern subspecies.

Over all investigated DNA markers, genetic variation was very low between the two *C. obscurus* subspecies, which might be due to recent divergence or interbreeding between the two subspecies. Hybridization, as a result of recent increase in population size and extension of habitats was discussed as the cause of low genetic differentiation between two NZ endemic Oystercatcher species (*Haematopus finschi* and *H. unicolor*), which also differ substantially in plumage, behavior and other measurements [19], [83], [84]. However, interbreeding between the two New Zealand Dotterel subspecies has not been documented [12], [13].

In summary, our analysis revealed a small degree of genetic divergence between the northern and southern populations, which is consistent with the morphological and behavioral characters that distinguish the two subspecies. We therefore support conservation efforts for both subspecies.

## Supporting Information

Figure S1 **Bootstrap Support and Bayesian Posterior Probabilities for Garli, BEAST and MrBayes analyses.** Illustration of topology differences between the three methods within the CRD II group.
(EPS)

Table S1 **All markers included in the study with GenBank/The Barcode of Life Database accession numbers.**
(PDF)

Table S2 **Substitution models for each partition used in Garli and MrBayes phylogenetic analyses.**
(PDF)
