# Supplementary material for: Phylogenetic Position and Subspecies Divergence of the Endangered New Zealand Dotterel (Charadrius obscurus)
Source: PLoS One. 2013 Oct 25;8(10):e78068. doi: 10.1371/journal.pone.0078068 (PMC3808304; doi:10.1371/journal.pone.0078068)
Supplement: Table S1 — All markers included in the study with GenBank/The Barcode of Life Database accession numbers. (PDF) [file pone.0078068.s002.pdf]

Group	Species	cytb	CO1	12s	16s	ND2	ND3	ATPase 6	ATPase 8	bFI7	RAG1
“Charadriiformes”: Charadriidae: Charadriinae: Anarhynchus	Anarhynchus frontalis	EF373118	BROM876-08	EF380263		EF373222					EF373167
“Charadriiformes”: Charadriidae: Charadriinae: Charadrius	Charadrius alexandrinus	AF417931	GQ481560				FR823167	FR822447	FR822447		
“Charadriiformes”: Charadriidae: Charadriinae: Charadrius	Charadrius alticola		FJ027340								
“Charadriiformes”: Charadriidae: Charadriinae: Charadrius	Charadrius asiaticus		BROM878-08								
“Charadriiformes”: Charadriidae: Charadriinae: Charadrius	Charadrius australis	EF373148	BROM542-07	EF373098		EF373256					EF373199
“Charadriiformes”: Charadriidae: Charadriinae: Charadrius	Charadrius bicinctus	AH003633	BROM726-07								
“Charadriiformes”: Charadriidae: Charadriinae: Charadrius	Charadrius collaris	AH005179	JN801556				FR823281			DQ881950	AY339106
“Charadriiformes”: Charadriidae: Charadriinae: Charadrius	Charadrius dubius		GU571329								
“Charadriiformes”: Charadriidae: Charadriinae: Charadrius	Charadrius falklandicus	AH005180	FJ027345								
“Charadriiformes”: Charadriidae: Charadriinae: Charadrius	Charadrius hiaticula		GU571330								
“Charadriiformes”: Charadriidae: Charadriinae: Charadrius	Charadrius leschenaultii		GQ481569								
“Charadriiformes”: Charadriidae: Charadriinae: Charadrius	Charadrius marginatus		BROM642-07				AM941567	FR822485	FR822485		
“Charadriiformes”: Charadriidae: Charadriinae: Charadrius	Charadrius melodus		DQ433491								
“Charadriiformes”: Charadriidae: Charadriinae: Charadrius	Charadrius modestus	AH005182	FJ027348				FM995617	FM995621	FM995621		
“Charadriiformes”: Charadriidae: Charadriinae: Charadrius	Charadrius mongolus	AF417927	GQ481571								
“Charadriiformes”: Charadriidae: Charadriinae: Charadrius	Charadrius montanus	AH005183	AY666249					AY794551	AY794551		
“Charadriiformes”: Charadriidae: Charadriinae: Charadrius	Charadrius morinellus	EF373130	GU571332	EF373080		EF373238					EF373182
“Charadriiformes”: Charadriidae: Charadriinae: Charadrius	Charadrius nivosus						FR823175	FR822423	FR822423		
**“Charadriiformes”: Charadriidae: Charadriinae: Charadrius**	**Charadrius obscurus aquilonius**	this study	BROM747-07	this study						this study	
**“Charadriiformes”: Charadriidae: Charadriinae: Charadrius**	**Charadrius obscurus obscurus**	this study								this study	
“Charadriiformes”: Charadriidae: Charadriinae: Charadrius	Charadrius pallidus		BROM880-08								
“Charadriiformes”: Charadriidae: Charadriinae: Charadrius	Charadrius pecuarius		BROM882-08				FR823147	FR822397	FR822397		
“Charadriiformes”: Charadriidae: Charadriinae: Charadrius	Charadrius peronii						FR823168	FR822417	FR822417		
“Charadriiformes”: Charadriidae: Charadriinae: Charadrius	Charadrius ruficapillus	AH005186	BROM883-08				FR823156	FR822437	FR822437		
“Charadriiformes”: Charadriidae: Charadriinae: Charadrius	Charadrius semipalmatus	EU166986	DQ433495	EU167040		EU166929					
“Charadriiformes”: Charadriidae: Charadriinae: Charadrius	Charadrius veredus	AH005189	BROM752-07								
“Charadriiformes”: Charadriidae: Charadriinae: Charadrius	Charadrius vociferus	DQ485890	AY666173	DQ485792	DQ485830	DQ385082	DQ385099			AY695205	AF143736
“Charadriiformes”: Charadriidae: Charadriinae: Charadrius	Charadrius wilsonia		AY666175								
“Charadriiformes”: Charadriidae: Charadriinae: Elseyornis	Elseyornis melanops	EF373128	BROM405-06	EF373078		EF373236					EF373180
“Charadriiformes”: Charadriidae: Vanellinae: Erythrogonys	Erythrogonys cinctus	EF373129	BROM727-07	EF373079		EF373237					EF373181
“Charadriiformes”: Charadriidae: Charadriinae: Oreopholus	Oreopholus ruficollis	EF373146	FJ027923	EF373096		EF373254					EF373197
“Charadriiformes”: Charadriidae: Charadriinae: Phegornis	Phegornis mitchellii	EF373149	BROM439-06	EF373099		EF373257					AY228781
“Charadriiformes”: Charadriidae: Charadriinae: Pluvialis	Pluvialis dominica		DQ433964	DQ674562	DQ674600					AY695201	
“Charadriiformes”: Charadriidae: Charadriinae: Pluvialis	Pluvialis squatarola	EF373151	GQ482512	EF373101		HM640806					EF373202
“Charadriiformes”: Charadriidae: Charadriinae: Thinornis	Thinornis novaeseelandiae	EF373161	BROM464-06	EF373113		EF373271					EF373214
“Charadriiformes”: Charadriidae: Charadriinae: Thinornis	Thinornis rubricollis	AH005185	BROM687-07								
“Charadriiformes”: Charadriidae: Vanellinae: Vanellus	Vanellus chilensis	EF373163	FJ028552	EF373115		EF373274					AY228772
“Charadriiformes”: Charadriidae: Vanellinae: Vanellus	Vanellus miles	FJ499016	BROM662-07								
“Charadriiformes”: Charadriidae: Vanellinae: Vanellus	Vanellus resplendens		JQ176606	DQ674565	DQ674603					AY695206	
“Charadriiformes”: Charadriidae: Vanellinae: Vanellus	Vanellus Vanellus	JQ342156	JQ342134							EF552790	AY339126
“Charadriiformes”: Haematopodidae: Haematopus	Haematopus finschi	EF514927	ROMC033-06	EF514931							
“Charadriiformes”: Haematopodidae: Haematopus	Haematopus palliatus		AY666233	DQ674563	DQ674601					AY695204	
“Charadriiformes”: Haematopodidae: Haematopus	Haematopus unicolor	EF514929	ROMC460-07	EF514933							
