# Supplementary material for: Phylogenetic Position and Subspecies Divergence of the Endangered New Zealand Dotterel (Charadrius obscurus)
Source: PLoS One. 2013 Oct 25;8(10):e78068. doi: 10.1371/journal.pone.0078068 (PMC3808304; doi:10.1371/journal.pone.0078068)
Supplement: Table S2 — Substitution models for each partition used in Garli and MrBayes phylogenetic analyses. (PDF) [file pone.0078068.s003.pdf]

	BIC *1	ratematrix =	statefrequencies =	ratehetmodel =	numratecats =	BIC *2	ratematrix =	statefrequencies =	ratehetmodel =	numratecats =
**12s**	TIM2+G	(0 1 0 2 1 2)	estimate	gamma	4	HKY	2rate	estimate	gamma	4
**16s**	TIM2+G	(0 1 0 2 3 2)	estimate	gamma	4	GTR	6rate	estimate	gamma	4
**mt_cp1**	TIM2+G	(0 1 0 2 3 2)	estimate	gamma	4	GTR	6rate	estimate	gamma	4
**mt_cp2**	HKY+G	2rate	estimate	gamma	4	HKY	2rate	estimate	gamma	4
**mt_cp3**	TIM3+G	(0 1 2 0 3 2)	estimate	gamma	4	GTR	6rate	estimate	gamma	4
**bFI7**	HKY	2rate	estimate	none	1	HKY	2rate	estimate	none	1
**RAG1_cp1**	HKY	2rate	estimate	none	1	HKY	2rate	estimate	none	1
**RAG1_cp2**	HKY	2rate	estimate	none	1	HKY	2rate	estimate	none	1
**RAG1_cp3**	K80+G	2rate	equal	gamma	4	K80	2rate	equal	gamma	4
	*1 jmodeltest settings for GARLI: 11 substitution schemes +F +G, nCat4, fixed BIONJ-JC									
	*2 jmodeltest settings for MrBayes: 3 substitution schemes +F +G, nCat4, fixed BIONJ-JC									
